# Pitch Perception in the First Year of Life, a Comparison of Lexical Tones and Musical Pitch

**DOI:** 10.3389/fpsyg.2017.00297

**Published:** 2017-03-09

**Authors:** Ao Chen, Catherine J. Stevens, René Kager

**Affiliations:** ^1^Utrecht Institute of Linguistics, Utrecht UniversityUtrecht, Netherlands; ^2^Communication Science School, Beijing Language and Culture UniversityBeijing, China; ^3^The MARCS Institute for Brain, Behaviour and Development, Western Sydney University, SydneyNSW, Australia

**Keywords:** lexical tone, musical pitch, perception development, cross-domain cognition, infancy

## Abstract

Pitch variation is pervasive in speech, regardless of the language to which infants are exposed. Lexical tone is influenced by general sensitivity to pitch. We examined whether the development in lexical tone perception may develop in parallel with perception of pitch in other cognitive domains namely music. Using a visual fixation paradigm, 100 and one 4- and 12-month-old Dutch infants were tested on their discrimination of Chinese rising and dipping lexical tones as well as comparable three-note musical pitch contours. The 4-month-old infants failed to show a discrimination effect in either condition, whereas the 12-month-old infants succeeded in both conditions. These results suggest that lexical tone perception may reflect and relate to general pitch perception abilities, which may serve as a basis for developing more complex language and musical skills.

## Introduction

The perceptual reorganization hypothesis assumes that acquiring native phonology involves learning the specific phonemic contrasts present in the to-be-learned language, whereas sensitivity to non-native contrasts gradually decreases. Such perceptual tuning occurs in the second half of the 1st year (Werker and Tees, 1984; Kuhl et al., 1992). Yet previous studies disagree on how the perception of lexical tones, or pitch contours realized on single syllables, changes in the 1st year of life. It is widely agreed that infants are highly sensitive to speech prosody (e.g., Mehler and Christophe, 1995; Nazzi et al., 1998; Soderstrom et al., 2011; Frota et al., 2014). With regard to lexical tones, several studies have found supportive evidence for such a decline in discrimination among non-tone language learning infants between 4 and 9 months (Harrison, 2000; Mattock and Burnham, 2006; Mattock et al., 2008; Yeung et al., 2013). Other studies, however, have found that sensitivity to lexical tones is maintained beyond the presumed perceptual reorganization window. Liu and Kager (2014) found that from 4 months onward, up until 17–18 months, Dutch infants were able to discriminate Chinese high-level and falling tone. When the acoustical distance between the two tones was reduced through manipulation, no discrimination was found between 9 and 15 months, yet the 5- and 17–18-month-olds succeeded at discrimination. English learning 14-month-old infants are able to learn words that are solely distinguished by lexical tones, and by 19 months, they are still able to discriminate Chinese rising and falling tones (Quam and Swingley, 2010; Hay et al., 2015). In addition, although it is a fact that non-tone language speakers find lexical tones notoriously difficult (Kiriloff, 1969; Bluhme and Burr, 1971; Shen, 1989), they can be fairly accurate at discriminating them (Burnham et al., 1996, 2015; So and Best, 2010; Chen et al., 2015). Non-tone language listeners’ acoustical sensitivity to lexical tones cannot simply reflect the effect of “nativeness,” but possibly sensitivity to pitch in language in general. Regardless of the salience of lexical tones, native tone language learning infants do not fully acquire lexical tones until childhood, and global intonation contours interfere with the recognition of lexical tones (Singh and Chee, 2016; Singh and Fu, 2016). In addition, although lexical tones are phonemic in Chinese, when learning novel words, 3-year-old Chinese children are more tolerant to lexical tone than to vowel mispronunciations (Ma et al., 2017). In sum, lexical tone perception seems flexible and exhibits a complex course of development.

It has been long debated whether language ability reflects domain specific mechanisms or whether it is the product of domain general development (e.g., Piaget, 1926; Fodor, 1983; Chomsky, 1986; Pinker, 1994; Tomasello, 2003). Language and music, two types of uniquely human sophisticated functions, are often compared to understand this question. Language and music are parallel in many aspects (Trehub, 2003). For both, pitch plays a fundamental role, and pitch contour (i.e., the shape of pitch patterns) forms a salient cue in perception (Yip, 2002; Trehub and Hannon, 2006). In the language domain, cross-linguistically, at phrase and sentence level intonation is largely encoded by pitch contour. Questions are commonly realized with a rising pitch contour whereas statements often carry a falling contour (e.g., Gussenhoven, 2004). Emphasizing certain aspects of information in many language or “focus” is often realized by raising pitch of the emphasized part and compressing pitch of the following part (Xu, 2011). In tone languages, lexical tones are used in a phonemic way to distinguish meaning at the lexical level (Yip, 2002). In music, pitch relations (rather than specific pitch levels where these relations are exhibited) are central for music perception and also play a role in memory. For example, for the vast majority of listeners, the same song played at a different pitch level is readily recognizable (e.g., Trehub and Hannon, 2006; Trainor and Hannon, 2013). In addition, adults are more sensitive to differences of “global contour” (i.e., the pattern of ups and downs) of melodies than to “intervals” (i.e., exact pitch distance between notes; e.g., Cuddy and Cohen, 1976; Dowling, 1978; Bartlett and Dowling, 1980; Schiavetto et al., 1999).

Although some pitch processing skills have been argued to be music specific (Hauser and McDermott, 2003; Peretz and Coltheart, 2003; Peretz et al., 2003), many studies have found positive correlations between pitch perception in both language and music domains, which suggests domain general cognitive mechanisms in pitch processing (e.g., Wong and Perrachione, 2007; Wong et al., 2012; Bidelman et al., 2013, among many others). Speaking a tone language natively modulates neural response to non-speech pitch (e.g., Chandrasekaran et al., 2007; Bidelman et al., 2011).

For music processing, the encoding of pitch contour is visible from very early on. Infants as young as 2 months are able to discriminate familiar and novel songs (Plantinga and Trainor, 2009), and by 6 months (and like adults), infants discriminate between songs by attending to the pitch contour rather than to specific pitch levels that they are played (Trainor et al., 2004; Plantinga and Trainor, 2005). Eight- to 11-month-old infants are sensitive to both contour-violating and contour-non-violating note changes, yet contour violation has been found to be perceptually more salient for infants than contour-sharing interval differences (Trehub et al., 1984, 1987). Moreover, infants are able to extract abstract pitch contour from the absolute pitch level at which it is played (Cohen et al., 1987; Trainor and Trehub, 1992). It should be noted that although infants discriminate songs from very early on (Trainor et al., 2004; Plantinga and Trainor, 2005, 2009), the songs not only differed in contour but also in rhythmic and temporal information. When using manipulated stimuli exhibiting contour differences alone, discrimination has only been attested on samples of infants older than 6 months (Trehub et al., 1984, 1987; Trainor and Trehub, 1992). It remains unknown whether younger infants are also sensitive to contour violation.

Although shared processing of lexical tone and music processing has been widely investigated among adults, not much is known regarding whether pitch perception development is related in these two domains in infancy. Mattock and Burnham (2006) tested both tone (Chinese and Cantonese) and non-tone (English) language learning infants on their discrimination of Thai tones as well as violin analogs of the tones. For the lexical tones, a decline of sensitivity was observed between 6 and 9 months among the English infants, but not among the Chinese infants. For the violin stimuli, however, both groups succeeded in the discrimination at both ages. By 10 months, native Japanese infants’ brain responses to pitch accents realized on words and to pure tones whose fundamental frequency was extracted from these words showed different lateralization patterns (Sato et al., 2010). These findings suggest that pitch perception develops in a domain specific manner. However, Mattock and Burnham (2006) and Sato et al. (2010) tested infants with *non-speech* rather than *musical* stimuli, as the analogs of lexical tones did not have a musical structure. The non-speech stimuli have no real life function, yet pitch contour is essential for perception and appreciation of music. In addition, these studies assume that lexical tones (or pitch accents) are *phonological* for infants, although non-tone language listeners may simply perceive them as *musical* (Chen et al., 2016).

In the current study, we investigate whether development observed in lexical tone perception may reflect general sensitivity to pitch, in the current study. We tested Dutch 4- and 12-month-old infants on their discrimination of lexical tones and comparable three-note musical melodies, both differing in pitch contour. A non-native pitch contrast was chosen so that the developmental change cannot be attributed to learning the specific tonal exemplars, and the music stimuli were manipulated so as to share similar properties to the lexical tones. We chose 4- and 12-month-olds since these age groups precede and follow perceptual reorganization, which allows us to observe whether development in lexical tone perception is language specific. As Dutch infants have shown high sensitivity to the contrast of Chinese high-level and high-falling tone (Liu and Kager, 2014) and to prevent a ceiling effect, we used two perceptually similar lexical tones (Hume and Johnson, 2001; Ma et al., 2017), namely the Chinese rising and dipping tones as the stimuli. Since, we focus on acoustic perception that underlies music and language processing, the infants were tested on their discrimination of single tokens of lexical tones and musical melodies, which prevented possible interference from normalization (Singh et al., 2004; Singh, 2008; Shi, 2010; Chen and Kager, 2015). If pitch contour perception develops in a domain general way, then we would expect a similar trajectory in both domains, possibly age-related enhancement. On the other hand, if development occurs in a domain specific manner, then based on the perceptual reorganization hypothesis (Mattock and Burnham, 2006; Mattock et al., 2008; Yeung et al., 2013) we would expect the 12-month-olds to be less sensitive than the 4-month-olds to the lexical tones, as these are linguistically irrelevant for the Dutch infants. For the musical stimuli, and given the high sensitivity to musical pitch contour among adults, a maintained or enhanced discrimination of the musical melodies should be observed.

## Materials and Methods

### Participants

One hundred and one infants were included in the analysis. All the infants were healthy full-term monolingual Dutch infants. There were 54 4-month-old infants (age range 4:01–4:29), 28 (18 boys, 10 girls) in the lexical tone condition and 26 (13 boys, 13 girls) in the music condition. There were 47 12-month-old (age range 12:01–12:29) infants, 23 in the lexical tone condition (10 boys, 13 girls), and 24 in the music condition (16 boys, 8 girls). Another 17 4-month-old infants were tested but excluded from analysis due to crying (*N* = 2), fussiness (*N* = 4), equipment failure (*N* = 1), experimenter error (*N* = 1), and failure to meet habituation criterion (*N* = 9, see below). Another 27 12-month-old infants were excluded from analysis due to crying (*N* = 7), fussiness (*N* = 4), equipment failure (*N* = 3), experimenter’s error (*N* = 2), parental interferences (*N* = 2), and failure to meet habituation criteria (*N* = 9).

As the experiment was not invasive and was conducted in a natural environment, Utrecht Institute of Linguistics did not require ethical approval at the time that the experiment was conducted. The experiments were conducted in accordance to guidelines of Utrecht Institute of Linguistics and Helsinki Declaration. Written consents from caregivers were obtained for all participating infants.

### Stimuli

For the lexical tones, in order to prevent a ceiling effect (Liu and Kager, 2014), Mandarin Chinese rising tone (T2) and dipping tone (T3) were used as stimuli, as they have been found to be relatively difficult to discriminate (Hume and Johnson, 2001; Chen et al., 2015). We used /ma/ as tone-bearing syllable, as an initial nasal consonant ensured continuous pitch. A female Mandarin speaker recorded the two syllables. Then the pitch contours of naturally produced /ma2/ and /ma3/ were extracted by the software PRAAT (Boersma and Weenink, 2009). After normalizing the duration of these two contours (450 ms), the pitch contours of the T2 after time normalization were re-synthesized onto the original T3 syllable using the PSOLA method (Moulines and Laroche, 1995). Time-normalization ruled out the possibility of interference from duration as a potential confounding factor in the experiment. Five native Mandarin speakers listened to the stimuli and were all in agreement that all the stimuli sounded like natural, normal speech. As young infants have shown difficulties in normalizing variable tokens (Singh et al., 2004; Singh, 2008; Shi, 2010), we only used one single token of each tone to prevent improvement in normalization from being a confounding factor for any development observed. To ensure that the comparability between tasks, we did not transpose the melodies in the music condition.

**FIGURE 1:**
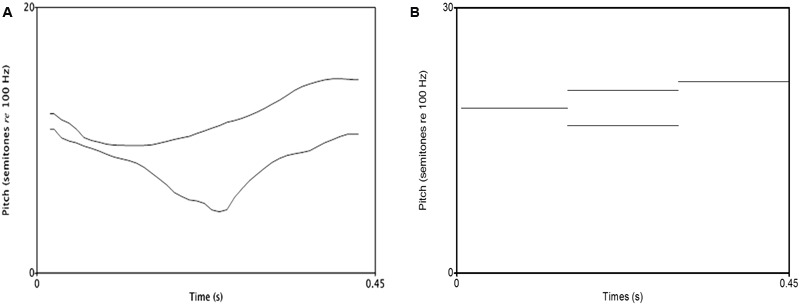
**Pitch contours of the rising and dipping tones used in the speech condition (A)** and those of the musical melodies **(B)**. Note that the first and last notes are the same in the two melodies.

For the musical melodies, 16th notes of D4, E4, F4, and C4 with a piano timbre were synthesized using a Nyquist script^1^^,^^2^. The notes were generated on the C4 (middle C) scale, along which the fundamental frequency of A4 equals 440 Hz, with the default duration (250 ms) of 16th notes in Nyquist. After synthesizing the four single notes separately, D, E, and F were concatenated to obtain a three-note rising melody— D-E-F, and D, C, and F were concatenated to obtain another three-note dipping melody— D-C-F. These two melodies were normalized to 450 ms and were then used as stimuli in this experiment. All the notes belonged to C major scale, which prevented possible discrimination based on key membership (Cohen et al., 1987). The two melodies had identical initial and final pitches, and the middle note determined global contour. This assured that the infants would not be able to discriminate the melodies by only attending to the onset or the offset. The difference between the two musical melodies was expected to be salient, as the middle note changed the pitch “direction” (e.g., up and down) rather than the “degree” of rising or falling (Trehub et al., 1984). The musical melodies and lexical tones had comparable contours, namely one rising and one dipping. **Figure 1** plots the pitch contours of the speech stimuli.

### Procedure

A visual habituation paradigm adapted from Liu and Kager (2014) was used, which has been found to be suitable for testing infants as young as 4 months. During the experiment, infants sat on their parent’s lap in the test cabin, and a 14-inch screen at the front displayed the visual stimuli, an infant-friendly colorful picture. The visual stimuli were contingent with the auditory stimuli, and the infants’ looking time to the visual stimuli was used as the indicator of their attention to the auditory stimuli. The auditory stimuli were presented at a comfortable volume through a frontal speaker. The parent listened to background music through headphones to prevent possible interaction with the infants. A hidden camera mounted above the screen recorded the infants’ looking behavior. The experimenter observed the video of the infants live and recorded whether the infant looked at the visual stimuli. For each trial, once the infant looked at the screen, the experimenter pressed a “looking” button on a button box to start the auditory stimuli. Whenever the infant looked away, the experimenter pressed another “non-looking” button on the same button box, and if the infant looked back to the screen, the experimenter pressed the “looking” button again. A trial ended if the infant looked away for more than 2 s, and an attention getter immediately appeared on the screen. Once the infant looked back at the screen, the experimenter started the next trial in the same way described above. The looking time of each trial as well as each look was automatically calculated on the experimenter’s computer.

The experiment consisted of a habituation and a test phase. Total looking time of the first three trials in the habituation phase was used as a baseline for measuring habituation. Starting from the fourth trial, the total looking time of each three consecutive habituation trials was calculated, and once this looking time was less than 65% of the total looking time of the first three habituation trials, the habituation criterion was met, and the test phase started automatically. The habituation phase had a minimum of six trials and a maximum of 12 trials. Those infants who failed to meet the habituation criterion within 12 trials were excluded from further analysis. The stimuli used for habituation were counter-balanced among the participants at each age for each condition. In the test phase, the infants were presented with one “old” trial, which was the same sound that they had heard in the habituation phase, followed by another “novel” trial, which was the new sound that they had not previously heard. In the test phase, if the infants were able to detect the difference between the two tones, then upon hearing the novel trial, their listening time should be recovered due to hearing something new. In both phases, a trial could have a maximum of 30 repetitions of the stimuli, with an inter-stimulus interval of 1 s. The same visual stimuli were used for the habituation and test. We did not counter-balance the order of test trials, and the current procedure was expected to highlight the discrimination response if there was any.

## Results

**FIGURE 2:**
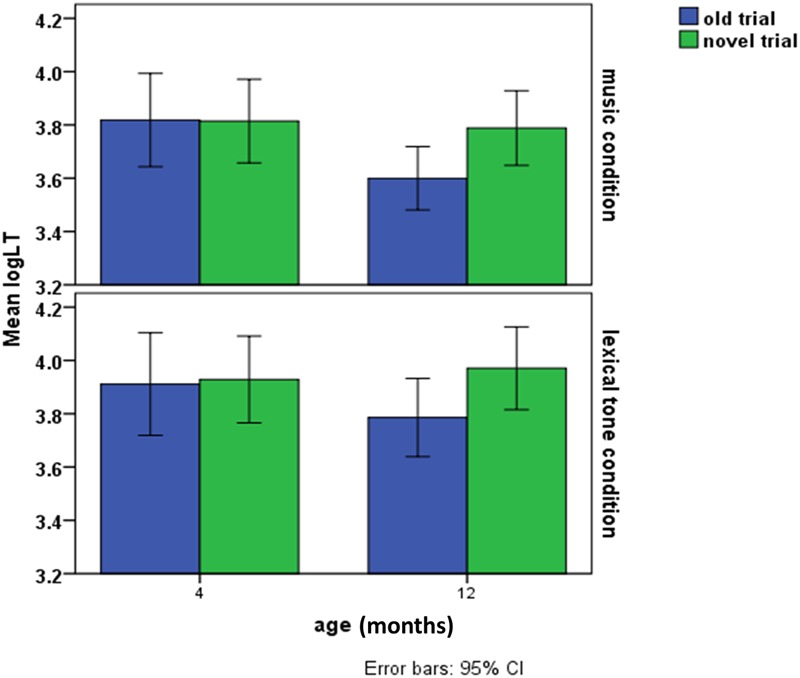
**LogLT of the old and novel trial in the lexical tone and music condition as a function of infant age**.

**Table 1** lists the raw looking time in the habituation phase and test phase in both conditions by both age groups. Before the analysis of test trials, infants’ response in the habituation phase was examined. A univariate ANOVA, taking condition and age as independent variables found a significant main effect of age, *F*(3,97) = 6.48, *p* < 0.05 (partial η^2^ = 0.063), where 4-month-olds needed more time to reach the habituation criterion. Condition, on the other hand, showed no significant effect, *F*(3,97) = 0.89, n.s.. No significant interaction between age and condition was found, *F*(3,97) = 0.002, n.s.. These findings suggest comparable habituation patterns for the music and the lexical tone condition. Next, the raw looking time of the infants was log transformed (base 10) to correct for skew (Gomez and Gerken, 1999; Gao et al., 2011). The log transformed looking times (logLT) of both age groups to both trial types fit a normal distribution. A repeated measures ANOVA was carried out with the logLT, where trial type (old/novel) was the within-subject factor, and condition (music/speech) and age (4/12-month-old) were between-subject factors. Trial type as well as condition showed a significant main effect *F*_trialtype_(1,97) = 5.20, *p* < 0.05 (partial η^2^ = 0.051); *F*_domain_(1,97) = 4.84, *p* < 0.05 (partial η^2^ = 0.047). A main effect of age was not significant, *F*_age_(1,97) = 1.58, n.s.. A significant interaction was found between age and trial type *F*(1,97) = 4.50, *p* < 0.05 (partial η^2^ = 0.044). *Post hoc* analyses found that, after merging domains only the 12-month-old infants showed a significantly longer logLT to the novel trial, *t*(46) = -2.88, *p* < 0.05. No other interaction was found to be significant. **Figure 2** depicts the logLT of the infants in each condition. As can be seen, for the 4-month-olds, no increase in listening time was observed for the novel trial in either condition. Such an increase, however, was found for the 12-month-old group in both conditions. The main effect of trial type was mainly driven by the 12-month-olds. In addition, both age groups had longer looking times in the lexical tone condition.

**Table 1:** Mean habituation time (s) and mean number of trials needed for habituation; raw looking time (s) to old and novel trial, and mean number of tokens in old and novel trial, separated by age group and condition.

			Music condition		Lexical tone condition	
			4 m	12 m	4 m	12 m
Habituation		Total time	107.01 (94.18)	72.61 (51.67)	120.59 (65.86)	85.00 (47.13)
		No. of trials	7.35 (1.68)	7.50 (1.96)	7.32 (2.02)	6.83 (1.16)
Test	old trial	Time	10.15 (9.26)	4.88 (3.51)	13.84 (12.60)	8.16 (6.71)
		Tokens	11.42 (7.53)	6.29 (2.84)	13.43 (10.25)	8.91 (5.27)
	novel trial	Time	9.73 (9.74)	8.02 (6.00)	12.99 (12.43)	12.35 (8.96)
		Tokens	10.54 (7.98)	9.00 (4.68)	12.57 (8.56)	12.00 (6.65)

*Numbers in brackets are standard deviations. Time was measured in seconds.*

## Discussion

In the current study, we investigated whether development in lexical tone perception may develop in parallel with perception of pitch in other cognitive domains namely music. The 4-month-olds did not show a discrimination effect in either the lexical tone or the music condition. For the lexical tones, at the age of 4 months, which has been assumed to precede the perceptual reorganization of lexical tones (Mattock and Burnham, 2006; Mattock et al., 2008; Yeung et al., 2013), Dutch infants failed to show a discrimination effect. Importantly, without inter-token variation, presumably the infants did not need to represent the lexical tones as *phonological categories*, but only needed to discriminate the lexical tones *acoustically*. The lack of a discrimination effect suggests that the 4-months-old infants did not perceive the acoustic difference between the two lexical tones. Similarly, without transpositions, the infants did not need to equalize the pitch contours played at different pitch levels before they could detect the contour violation, yet no discrimination was found. It is likely that the skills that adult listeners readily make use of when processing music are not fully mature at the beginning of life (Dowling, 1978; Schiavetto et al., 1999). The lack of discrimination effect in both conditions suggests that at 4 months, the infants are not proficient at processing the acoustic attributes that are exploited by linguistic and musical structures.

By 12 months, a parallel enhancement was observed in both the music and the language conditions. Importantly, what we show in the current study is that language input may not be the only factor driving perceptual development, and the perceptual behavior elicited by linguistic stimuli may reflect a general auditory rather than language specific development. As the infants were not exposed to lexical tones in their ambient input, the improvement cannot be explained by learning the lexical tones *per se*, but must reflect a general ability in dealing with pitch in speech. The similar developmental trajectory in both domains suggests that improved auditory pitch acuity may form a common basis for developing cognitively more advanced skills in language and music. The enhanced pitch perception may correlate with auditory maturation. Although frequency tuning is mature at birth at the cochlea level (Abdala et al., 1996), frequency resolution becomes adult-like between 3 and 6 months (Spetner and Olsho, 1990). Auditory brainstem also matures within the first 6 months after birth, and the maturation of auditory cortex continues to childhood (see Moore and Linthicum, 2007 for a review). At this moment, it is hard to infer whether the processing of musical and speech pitch recruited the same neural resources within the sample, yet basic auditory abilities seem to develop in a domain-general fashion. The physiological basis for successful discrimination of pitch realized on ecologically valid and spectrally complex sounds needs further investigation. It would be interesting for further study to investigate how such improved perception contributes to higher level processing such as phonological categorization or representation of musical pitch contours across pitch levels and musical instruments, and whether these abilities also show a comparable developmental trajectory in language and music.

So far, the perception of non-native lexical tones has been mostly studied in infants between 6 and 9 months (Harrison, 2000; Mattock and Burnham, 2006; Mattock et al., 2008; Yeung et al., 2013), and lexical tones are considered to be non-native *phonological* contrasts for infants learning a non-tone language. Pitch variation, however, is a language universal. The need to distinguish and understand intonation may help infants improve their sensitivity to pitch in general, which is reflected in their discrimination of lexical tones. It is possible that the 12-month-old Dutch infants assimilated T2 to a salient pitch contour in Dutch question rise. Non-tone language adults have been found to maintain a high psycho-acoustically based perceptual sensitivity to non-native lexical tones (Burnham et al., 1996, 2015; So and Best, 2010; Chen et al., 2015). Non-native infants’ sensitivity to lexical tones can remain after the assumed perceptual organization window (Liu and Kager, 2014; Chen and Kager, 2015; Hay et al., 2015). In the current study, we used a perceptually similar contrast than those used in Liu and Kager (2014; Hume and Johnson, 2001), and a progression from 4 to 12 months was observed. A growing body of evidence shows that the perception of speech sounds does not follow a single developmental trajectory (Narayan et al., 2010; Liu and Kager, 2014; Mazuka et al., 2014; Tsuji and Cristia, 2014; Tyler et al., 2014), and infants do not completely lose sensitivity to non-native contrasts. Our results, together with these other studies, lead to the question of what underlies perceptual attunement. It is possible that when infants grow older, they become less capable of perceiving non-native contrasts *phonologically*, but at the same time, psycho-acoustical perception may improve. Yet whether a better auditory perception can be found in general for speech sounds after 9 months, or whether such improvement is restricted to certain types of speech sounds, such as vowels (Mazuka et al., 2014) and pitch, needs further investigation. Perceptual narrowing is well motivated given the need to efficiently process environmentally relevant distinctions (Scott et al., 2007) and by observations that adults cannot learn a language as easily as infants. The inability to perceive non-native contrast has been claimed to be one of the hindrances to proficient learning in adults. Yet more efforts should be made to understand what exactly complicates non-native language perception and when exactly we lose the ease to perceive non-native contrasts.

In the music domain, sensitivity to contour differences has been claimed to be visible from very early on (Plantinga and Trainor, 2009; Stefanics et al., 2009). However, Plantinga and Trainor (2009) tested 2-month-old infants with songs, and such discrimination only called for coarse representation of the melodies, as the songs differed from one another on multiple dimensions, including rhythm and tempo. Our task, on the other hand, tested the detection of contour violation with manipulated stimuli, and the 4-month-olds failed. Hence, it is possible that young infants are able to coarsely represent pitch contours, yet their accurate perception of pitch details is still under-developed. In our task, the middle note violated the contour, and the edge notes were not informative. Several studies have proposed an “edge benefit” in rule learning, namely that the edge serves as the anchoring position, and items in a stream are memorized relative to the edge item (Hitch, 1996; Henson, 1998; Endress et al., 2005). It may be the case that young infants have difficulties perceiving pitch change at a medial position, which may hinder them in noticing the change of contour efficiently. It would be interesting for future studies to test whether young infants could more easily detect a contour violation occurring at an edge position.

Finally, it should be acknowledged that our musical stimuli were generated to match the lexical tones. The constituent notes had a slightly shorter duration compared to previous studies (e.g., Trainor and Trehub, 1992). It might be the case that for the younger group, the short duration hindered the infants from sufficient representation of each individual note, where the violation of contour was realized. When presented with the same stimuli, the 12-month-olds did show a clear discrimination effect. This suggests that the better contour violation perception at 12 months may be due to a higher temporal resolution in auditory perception (Morrongiello et al., 1984; Werner et al., 1992). Nevertheless, our musical stimuli were ecologically valid, as a 16th note has a duration of 125 ms when the tempo is 120 beats-per-minute. In addition, our stimuli were highly representative of pitch in speech and pitch in music: the musical ones were composed of discrete notes without segmental information, whereas the lexical tones had continuous pitch contours and were realized on syllables. Therefore, the distinction between music and speech stimuli was still maintained, and it is convincing that infants show a general enhancement in auditory pitch perception in the 1st year of life.

## Conclusion

In the current study, we tested Dutch 4- and 12-month-old infants on their discrimination of pitch contours realized in speech, specifically, the Chinese rising and dipping tones, as well as musical stimuli exhibiting analogous pitch contours. We found that the 4-month-olds failed to show discrimination in either condition, whereas the older group succeeded in both conditions. These findings suggest that pitch perception develops in a domain-general fashion in early infancy, and development in speech perception may reside in more general auditory enhancement, and may not be a language specific development.

## Author Contributions

AC contributed to the design of the work, acquisition and analysis of the data and drafting the work. CS contributed to the interpretation of the data, drafting and revising the work. RK contributed to the design of the work, interpretation of the data, drafting and revising the work.

## Conflict of Interest Statement

The authors declare that the research was conducted in the absence of any commercial or financial relationships that could be construed as a potential conflict of interest.

## Footnotes

1 http://audacity.sourceforge.net/help/nyquist

2 http://www.cs.cmu.edu/~music/music.software.html
